# New Insights into Pacing Induced Cardiomyopathy

**DOI:** 10.31083/j.rcm2504118

**Published:** 2024-03-27

**Authors:** Sung Soo Kim, Hyung Wook Park

**Affiliations:** ^1^Department of Cardiovascular Medicine, Chosun University Medical School, 61452 Gwangju, Republic of Korea; ^2^Department of Cardiovascular Medicine, Chonnam National University Medical School, 61469 Gwangju, Republic of Korea

**Keywords:** pacemaker, artificial, heart failure

## Abstract

Pacing induced cardiomyopathy (PICM) can occur as a complication due to pacing the right ventricle. Its precise definition varies across different studies, leading to uncertainty as to the best approach for managing this entity. More than 10% of patients who undergo chronic right ventricular pacing develop PICM. Risk factors associated with PICM include reduced left ventricular ejection fraction (LVEF), the proportion of right ventricular pacing, and paced QRS duration. The main approach to treating PICM has been upgrading to biventricular pacing cardiac resynchronization therapy when the LVEF decreases. However, emerging evidence suggest that conduction system pacing might provide an opportunity to manage PICM.

## 1. Introduction

Since the introduction of cardiac pacing in 1958, the right ventricle (RV) has been the favored site for implanting permanent pacemaker (PPM) leads, attributed to considerable expertise, ease of implantation, and the stability provided by passive fixation leads within the trabeculae of the RV [1]. Nevertheless, extended periods of RV pacing are linked to progressive left ventricular (LV) dysfunction, caused by asynchronous activation of the ventricles, leading to significant functional, hemodynamic, electrical, and structural alterations [2, 3]. In some cases, LV function may deteriorate following PPM implantation without a clear cause, a condition referred to as pacing-induced cardiomyopathy (PICM). PICM is typically characterized by a reduction in left ventricular ejection fraction (LVEF) in patients with a high burden of RV pacing and no other identifiable cause. It has been reported that 10–20% of patients develop PICM after 2–4 years of RV pacing [4]. PICM is associated with an increased risk of developing atrial fibrillation (AF) [5, 6], hospitalization due to heart failure (HF) [3, 5, 7], and cardiac mortality [3, 5, 7, 8, 9]. In patients with PICM, upgrading to biventricular pacing cardiac resynchronization therapy (BiV-CRT) has been shown to alleviate HF-related symptoms and promote reverse remodeling of the LV. Recently, conduction system pacing (CSP), such as His bundle pacing (HBP) and left bundle branch area pacing (LBBAP), have demonstrated substantial improvements in LVEF and HF symptoms in patients with PICM.

## 2. Definition 

A significant number of patients with a normal pre-implant LVEF who require RV pacing are prone to developing PICM. PICM is characterized by a decrease in LVEF and the emergence of symptoms associated with systolic heart failure. Although there is no single universally accepted definition of PICM, the current guidelines [10] recommend that it should meet the following criteria:

1. A decrease in LVEF of at least 10%, starting from a baseline LVEF above 50% prior to RV pacing.

2. Substantial RV pacing (pacing percentage >20%).

3. No other definitive cause for reduction in LVEF following RV pacing.

**Fig. 1.**
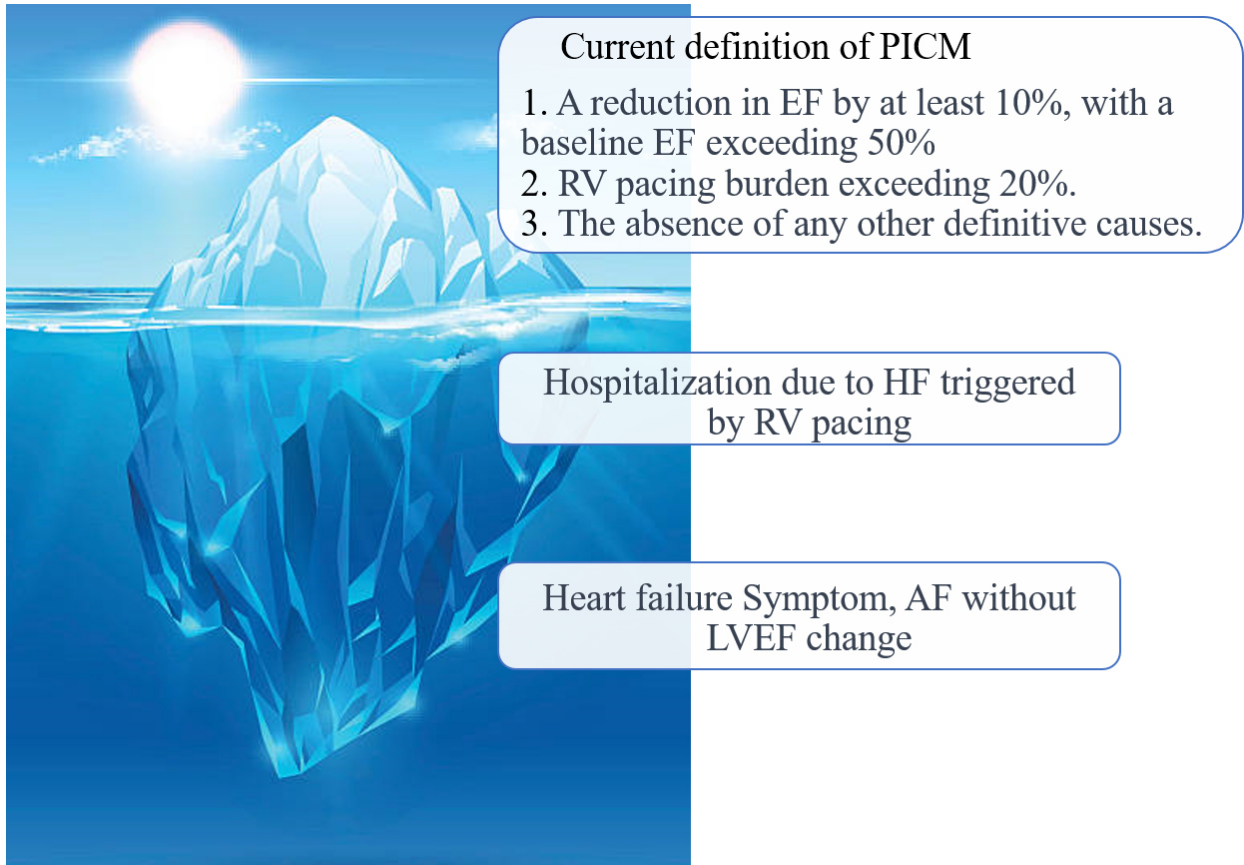
**Definition of pacemaker induced cardiomyopathy**. PICM, pacing-induced cardiomyopathy; EF, ejection fraction; RV, right ventricle; HF, heart failure; AF, atrial fibrillation; LVEF, left ventricular ejection fraction.

In some studies, the definition of PICM included heart failure symptoms and hospitalization (Fig. 1) [4, 11]. This inclusion is particularly relevant because patients with HF resulting from RV pacing may still have a relatively preserved LVEF. In the pacing to avoid cardiac enlargement (PACE) study, 177 patients exhibiting a normal initial EF were designated to undergo either Biventricular Pacing (BVP) or Right Ventricular Pacing (RVP). The mean EF of patients at the start of the study was 61.7% [12]. After 12 months of follow-up, the mean LVEF dropped to 54.8% in the RVP cohort but stayed constant at 62.2% in the BVP cohort (*p* < 0.001). Despite the LVEF maintaining within the normal range, the decline in LVEF within the RVP group correlated with a notable rise in left ventricular end-systolic volume (LVESV). Throughout the 4.8-year average follow-up [7], the two cohorts exhibited continued divergence, with a further dip in LVEF in the RVP group to an average of 53.2% and an ongoing escalation in LVESV. In contrast, these parameters remained steady in the BVP group. Additionally, despite the relatively moderate reduction in LVEF within the RVP group, there was a notably elevated occurrence of heart failure hospitalization (HFH) in the RVP group (23.9% *vs.* 14.6%, *p* = 0.006).

The definition of pacemaker induced cardiomyopathy (PICM) varies among studies; however, current guidelines recommend defining PICM as a >10% decrease in LVEF after chronic RVP, resulting in an LVEF ≤50%, with the pacing percentage of RVP exceeding 20%. Nevertheless, it has been suggested that the incidence of PICM is significantly underestimated when defined solely by a reduction in LVEF. While assessing LVEF is crucial, the development of HF symptoms, HFH, and the onset of AF also plays a significant role in PICM.

These findings suggest that many patients who experience adverse effects from RV pacing may still have preserved EF and may not meet the conventional definition of PICM. While assessing LVEF remains crucial, the emergence of HF symptoms or the occurrence of HFH also plays a significant role in identifying PICM. Some patients may develop symptoms due to ventricular dyssynchrony and elevated cardiac filling pressures before a noticeable decline in LVEF becomes apparent. In certain cases, PICM may present as a form of heart failure with preserved EF, which is termed the PICM syndrome. Several studies support expanding the definition of PICM to encompass the onset of heart failure symptoms following PPM implantation, regardless of specific LVEF criteria [4, 11].

Besides the decline in LVEF and heart failure hospitalization, there is a suggestion that the onset of AF could also serve as an indication of PICM in certain patients. Nielsen *et al*. [6] observed that a greater burden of ventricular pacing in the dual-chamber pacing group significantly elevated the risk of AF over a 2.9-year follow-up compared to atrial-only pacing (23.3% *vs.* 7.4%, *p* = 0.03). Similarly, in the MOST (MOde Selection Trial) study, the occurrence of AF demonstrated a relatively linear increase with a higher burden of ventricular pacing. Nevertheless, it’s crucial to highlight the difficulties in precisely determining the occurrence of atrial fibrillation directly linked to extensive RV pacing, especially considering the complex interaction between cardiomyopathy and atrial arrhythmias.

## 3. Incidences

The incidence of PICM varies significantly depending on the chosen definition, but on the whole, it appears to affect approximately 10–20% of individuals within 3–4 years following permanent pacemaker insertion [12, 13, 14]. This variability can be attributed to variations in how PICM is defined, differences in the characteristics of the studied patient populations, and disparities in the duration of follow-up.

The study illustrating the impact of definition on the incidence of PICM within a single cohort was reported by Kaye *et al*. [14]. In this investigation, three distinct definitions for PICM were utilized: (1) EF ≤40%, if the baseline EF was ≥50%, or an absolute reduction in EF ≥5% if the baseline EF was <50%, (2) EF ≤40%, if the baseline EF was ≥50%, or an absolute reduction in EF ≥10% if the baseline EF was <50%, (3) an absolute reduction in EF ≥10%, regardless of the baseline EF. Over the 3.4-year follow-up period, the occurrence of PICM ranged from 9.3%, 5.9%, to 39.0% depending on the chosen PICM definition.

It is important to note that all of these studies on PICM were conducted retrospectively and had varying criteria for defining cardiomyopathy and the percentage of RVP as inclusion criteria, which makes them susceptible to selection bias. A systematic review of PICM studies estimated the incidence to be 12%, although the data was limited due to the variability in PICM definitions and the duration of follow-up across the studies [15].

## 4. Pathophysiology 

**Fig. 2.**
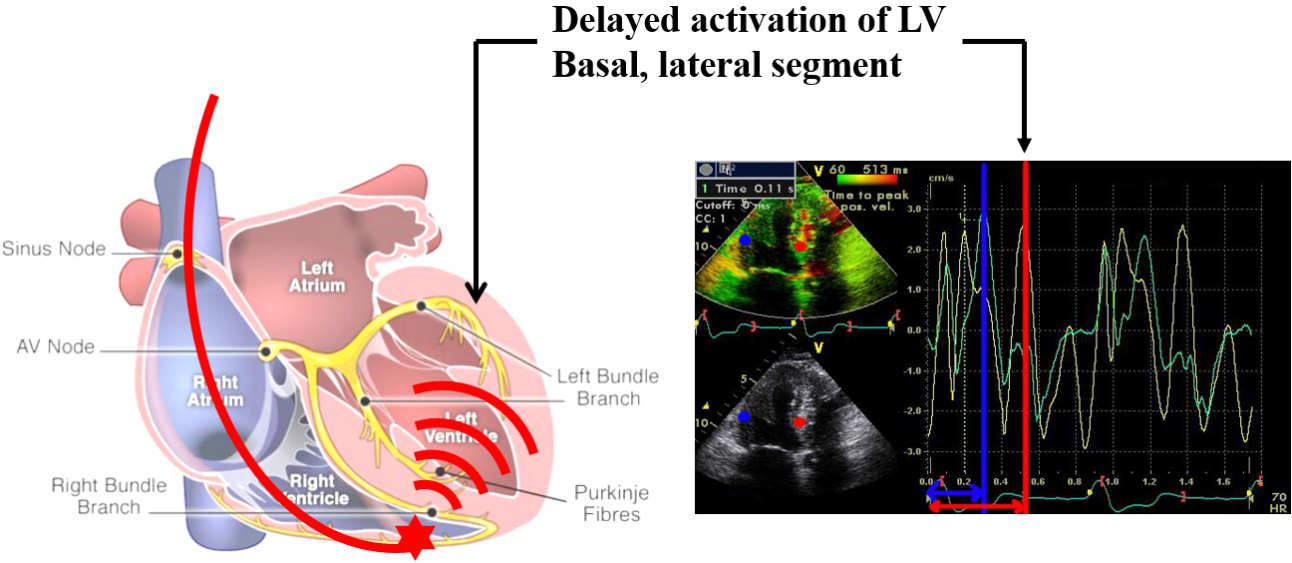
**Right ventricular pacing induced left ventricular dyssynchrony**. LV, left ventricular.

Although the specific pathophysiological mechanisms underlying the development of PICM have not been fully understood, it has been hypothesized that ventricular dyssynchrony plays a central role. In RV apical pacing, areas with early electrical activation exhibit early contraction, whereas segments of the left ventricle that activate late experience delayed contraction. This disparity in electrical activation timing between the RV and LV results in irregular mechanical contraction, commonly referred to as ventricular dyssynchrony (Fig. 2). In the absence of involvement of the His-Purkinje system, there is a sluggish transmission of electrical impulses from one myocyte to another, often characterized by a solitary point of activation across the ventricular septum. The latest activation typically takes place in the inferior, basal left ventricle [16]. The disturbed electrical activation leads to compromised mechanical contraction. Regions closest to the pacing site undergo rapid systolic shortening, leading to pre-stretching of late-activating areas. This process results in a redistribution of myocardial strain and workload, ultimately leading to less efficient overall contraction [17]. Redistribution of myocardial workload can also induce alterations in cardiac metabolism, giving rise to regional irregularities in myocardial blood flow. Long- term, RV pacing can affect cardiac function and clinical outcomes by altering cardiac histology, such as myocardial fibrosis in patients with congenital atrioventricular (AV) block after long-term RV pacing [18]. Consequently, some individuals subjected to prolonged RV pacing may develop cardiomyopathy resulting from dyssynchrony, leading to a reduction in left ventricular ejection fraction and the onset of heart failure. While this adverse remodeling is considered a chronic process that takes months or years to culminate in cardiomyopathy, changes in LVEF can be discerned within hours of RV pacing [19]. These acute effects align with clinical studies demonstrating a notable increase in heart failure incidence during the initial weeks to months of high-burden pacing.

In the context of pacing the RV at the apex, areas with early electrical activation exhibit early contraction, while segments of the LV that activate later experience delayed contraction. When the His-Purkinje system is not involved, there is sluggish myocyte-to-myocyte propagation, marked by a solitary breakthrough of activation across the ventricular septum. Typically, the latest activating site is found in the inferior, basal region of the left ventricle. The blue line represents the activation time of the septal wall, and the red line represents the activation time of the lateral wall.

The pathophysiology of PICM exhibits resemblances to other cardiomyopathies linked to impaired electrical conduction, such as left bundle branch block (LBBB) and premature ventricular contractions. Because of these shared characteristics, they are collectively known as dyssynchrony-associated cardiomyopathies [11]. The likelihood of developing cardiomyopathy increases with a higher frequency of dyssynchrony. In the case of PICM, an RV pacing burden of ≥20% is correlated with an elevated risk of developing PICM. Furthermore, there is evidence indicating that the reversal of cardiomyopathy may be achievable if the frequency of dyssynchrony is reduced [20].

## 5. Risk Factor

Several factors, including advanced age [21], male gender [22], atrial fibrillation [12], increased pacing burden [5], impaired LVEF [3], prolonged QRS duration [23, 24, 25, 26, 27], diastolic dysfunction [28] and abnormal global longitudinal strain [29] have been identified as independent predictors of the development of PICM.

Patients can tolerate a substantial burden of RV pacing for an extended period without experiencing noticeable adverse effects. The relationship between pacing-induced heart failure and PICM remains unclear, with inconsistent direct correlations observed in studies examining predictors of PICM. Furthermore, certain individuals with PICM may not develop heart failure, maintaining exercise capacities and quality of life comparable to those without PICM [30]. There is considerable variability in individual susceptibility to the deleterious effects of RV pacing, underscoring the need for additional research to pinpoint individuals at the highest risk of developing PICM and to customize preventive measures accordingly.

Previous studies have demonstrated that a lower EF is a statistically significant factor in the development of PICM [3, 5]. In the Dual Chamber and VVI Implantable Defibrillator (DAVID) trial [3], among individuals considered for defibrillator implantation, those with a more than 40% RV pacing burden exhibited an incidence of death or heart failure hospitalization exceeding 30% at 18 months, in contrast to less than 10% in those with lower RV pacing burdens. Similarly, in the MADIT II [31], an RV pacing burden surpassing 50% was associated with nearly twice the risk of new or worsening heart failure symptoms. High RV pacing burden can markedly worsen left ventricular dysfunction, even in patients with only mildly reduced baseline LVEF.

An RV pacing burden exceeding 40% has been correlated with a heightened risk of heart failure hospitalization, as evidenced in the MOST Trial [5]. In a comparison between VVI and DDD pacing in sinus node dysfunction, DDD pacing exhibited a heart failure hospitalization incidence almost 2.5 times higher among those with a greater than 40% RV pacing burden, compared with those with lower burdens of VVI. Substantial RV pacing is defined as pacing that either exceeds or is expected to exceed 40%. Nonetheless, some observational studies have suggested that RV pacing beyond 20% may also yield unfavorable consequences [22, 32].

RV pacing induces a left ventricular electrical activation pattern resembling left bundle branch block (LBBB), leading to electrical dyssynchrony and a prolonged QRS duration due to slow myocardial conduction. Consequently, a prolonged paced QRS duration is identified as a risk factor for PICM, indicating that patients with a lengthier paced QRS duration face a higher risk of developing PICM [23, 24, 25, 26]. Some researchers propose that a paced QRS duration of 150 ms is a sensitive indicator of PICM [24]. However, it’s crucial to note that while there is an association between paced QRS duration and PICM, there is no established causal relationship. A prolonged paced QRS duration might signify a higher degree of myocardial disease and an increased risk of left ventricular dysfunction, irrespective of RV pacing. Additionally, it may suggest that adverse remodeling due to dyssynchrony has already taken place.

A study has shown that diastolic dysfunction is a risk factor for PICM in patients with preserved LV function [28]. Diastolic function constitutes an equally crucial aspect of the cardiac cycle, intimately connected with systolic function. Impaired diastolic relaxation, filling, or distensibility of the LV, resulting from diastolic dysfunction, compromises LV contractility [33]. When diastolic dysfunction is present, the added stress induced by RV pacing might lead to further functional abnormalities, including electromechanical delay due to a pacing-induced left bundle branch block pattern and regional perfusion defects [34]. As a result, RV pacing has the potential to contribute to a heightened degree of LV systolic dysfunction and an increased likelihood of clinical heart failure.

Myocardial strain is an emerging parameter for a more detailed evaluation of the systolic function of cardiac chambers. Among strain parameters, global longitudinal strain has received the most scrutiny. It exhibits greater sensitivity than LVEF and can detect subclinical left ventricular dysfunction [35]. Recent research indicates that global longitudinal strain could function as a predictor for the deterioration of LV systolic function after pacemaker implantation, potentially identifying patients at risk for PICM [36].

## 6. Treatment

PICM might be reversible through enhancement of dyssynchrony. Hence, in addition to adhering to recommended medical therapy for heart failure with reduced ejection fraction, alternative pacing strategies have been suggested. These strategies encompass upgrading to BiV-CRT or adopting other more physiologically aligned pacing methods like HBP or LBBAP, which are linked to considerably reduced ventricular dyssynchrony [37].

The predominant approach for addressing PICM involves upgrading to BiV-CRT. Current guidelines advocate for patients with a cardiac implantable electronic device and a decline in left ventricular function or worsening heart failure symptoms due to substantial ventricular pacing to consider upgrading to BiV-CRT for enhanced LV function and relief from heart failure symptoms (Table 1, Ref. [20, 32, 38, 39, 40]). In a recent retrospective review involving 1279 consecutive BiV-CRT cases, 78 patients with PICM were identified [20]. The study indicated that BiV-CRT was highly successful in reversing PICM, with 86% of patients experiencing an improvement in LVEF by more than 5%. A prospective cohort study also demonstrated symptom alleviation and reversal of LV remodeling (LVESV decrease >15%) in patients who upgraded to BiV-CRT, as supported by a meta-analysis showcasing improvements in various aspects of heart function and quality of life [39]. A recent investigation showed that in comparison to an implantable cardioverter defibrillator (ICD), upgrading to cardiac resynchronization therapy-defibrillator (CRT-D) resulted in decreased morbidity and mortality, along with enhanced left ventricular reverse remodeling among patients with PICM [40]. The study encompassed 360 patients exhibiting symptoms of heart failure, reduced ejection fraction (≤35%), wide-paced QRS complex (≥150 ms), and a high burden of RV pacing (≥20%). These individuals were randomly assigned to either receive a CRT-D or an ICD. Additionally, all participants had previously received a pacemaker or ICD at least six months prior and were already undergoing guideline-directed medical therapy. The primary outcome was the composite of HF hospitalization, all-cause mortality, or <15% reduction of LV end-systolic volume, which occurred in 58 out of 179 patients (32.4%) in the CRT-D group and 101 out of 128 patients (78.9%) in the ICD group over a median duration of 12.4 months. The positive impact of upgrading to CRT-D remained consistent across all predefined subgroups. Furthermore, the combined incidence of heart failure hospitalization and all-cause mortality was lower in the CRT-D cohort compared to the ICD cohort. Additionally, the evaluation of left ventricular morphology and function using echocardiography favored CRT-D over ICD, with a 12-month disparity of –39.00 mL in left ventricular end-diastolic volume and a difference of 9.76% in left ventricular ejection fraction.

**Table 1.** **Upgrade to biventricular pacing cardiac resynchronization therapy in patients with pacemaker induced cardiomyopathy**.

Study	Year	Design	Total number of patients	Number of patients upgraded to BiV-CRT	Follow-up period (months)	Baseline EF (%)	Post BiV-CRT EF (%)	Paced QRS duration	Clinical outcomes
Merkely *et al*. [40]	2023	Multicenter, randomized comparative (CRT-D *vs.* ICD)	360	215	12	25	36		Improvement of all-cause mortality, HF hospitalization (*p* < 0.01)
Loboda *et al*. [38]	2020	Retrospective cohort	115	115	72	27	31	180	No difference in all-cause mortality
Khurshid *et al*. [20]	2018	Retrospective, cohort	1279	69	7	29	45	184	86% (LVEF improvement >5%)
Kiehl *et al*. [32]	2016	Retrospective, cohort	823	101	168	34	45	161	84% (LVEF >10% or LVESV decrease >15%)
Schwerg *et al*. [39]	2015	Prospective cohort	615	20	6	33	48	152	85% (LVESV decrease >15%)
									Improvement of NYHA class (*p* < 0.05)

BiV-CRT, biventricular pacing cardiac resynchronization therapy; EF, ejection fraction; HF, heart failure; LVESV, left ventricular end systolic volume; NYHA, New Work Heart Association; CRT-D, cardiac resynchronization therapy-defibrillator; ICD, implantable cardioverter defibrillator.

However, the upgrade from right ventricular pacing to BiV-CRT is limited by conditions related to venous vascular access and cardiac venous anatomy [41]. Challenges include a 5% to 7% probability of unsuccessful coronary sinus (CS) lead placement due to anatomical variations (CS valves, tortuosity, small-caliber target vessels), elevated pacing thresholds, and diaphragmatic stimulation [42]. Factors such as AV optimization, LV lead thresholds, and the choice of LV electrodes in quadripolar leads can also impact the response to BiV-CRT upgrades. The procedure entails an elevated risk of pocket or lead infection and the potential for LV pacing lead fracture or dislodgment [43, 44, 45]. In the National Inpatient Sample Database, Cheung *et al*. [43] found that CRT upgrade procedures were associated with cardiac perforation (1.3%), pneumothorax (1.3%), and lead revision (2.9%). Similar findings were found in the replace registry [44]. A prospective multicenter registry on complications related to cardiac implantable device replacement revealed that complications linked to device upgrades included cardiac arrest (0.3%), pneumothorax (0.8%), cardiac perforation or tamponade (0.7%), and lead-related issues (7.9%). The highest complication rates were noted in patients who underwent an upgrade or revision of CRT (18.7%, 95% confidence interval (95% CI) 15.1 to 22.6). These results underscore that, while upgrading to CRT is highly effective in PICM patients, the associated complications should not be underestimated.

Recent investigations have assessed the role of physiological pacing through His-Purkinje conduction system pacing (HPCSP), demonstrating significant enhancements in LVEF and HF symptoms in PICM patients. HBP has been proven to markedly reduce the risk of HF hospitalization compared to RV pacing (Table 2, Ref. [46, 47, 48]) [46, 47, 48, 49, 50]. In individuals with RV pacing, HBP resulted in a significantly narrower paced QRS duration and notable improvements in EF for those with PICM [46]. In comparison to BiV-CRT, one prospective cohort study revealed that HBP led to improvements in New York Heart Association (NYHA) class and LVEF after 6 months [47]. While HBP can reverse LV remodeling in PICM patients, its application is constrained by relatively low implant success rates and unstable pacing parameters (higher pacing thresholds, which may lead to premature battery depletion, lower sensing values, and reduced success rates in patients with bundle branch block) [48].

**Table 2.** **Upgrade to His bundle pacing in patients with pacemaker induced cardiomyopathy**.

Study	Year	Design	Total number of patients	Number of patients upgraded to HBP	Follow-up period (months)	Baseline EF (%)	Post HBP EF (%)	Pre HBP QRS duration (ms)	Post HBP QRS duration (ms)	Clinical outcomes
Shan *et al*. [48]	2018	Prospective cohort (HBP)	11	11	24	36	53	156	107	Improvement of NHYA
										Decreased BNP (*p* < 0.01)
Vijayaraman *et al*. [46]	2019	Retrospective, Case study (HBP *vs*. RVP)	85	79	25	34	48	123	114	Improvement of NHYA (*p* < 0.01)
Gardas *et al*. [47]	2022	Prospective (HBP *vs*. BiV-CRT)	61	39	6	34	48	182	118	Improvement of NHYA (*p* = 0.04)

BiV-CRT, Biventricular pacing cardiac resynchronization therapy; BNP, brain natriuretic peptide; EF, ejection fraction; HBP, His bundle pacing; NYHA, New Work Heart Association; RVP, right ventricular pacing.

LBBAP, initially introduced by Huang *et al*. in 2017 [51], has attracted growing interest as a novel physiological pacing technique in recent years. LBBAP entails the direct pacing of the left bundle branch via the transseptal approach and offers advantages such as enhanced R-wave amplitude, relatively lower thresholds, and a greater likelihood of correcting LBBB by pacing more distally to the site of conduction block [52, 53]. Although pacing thresholds and R-wave amplitudes were superior with LBBAP compared to HBP, they remained consistent during medium-term follow-up. While theoretically HBP might be considered superior to LBBAP due to complete interventricular synchrony, recent studies have demonstrated a reduced incidence of death and heart failure hospitalizations compared to RV pacing [54, 55, 56, 57, 58]. In small-sized retrospective studies, upgrading to LBBAP improved cardiac function and NYHA class in PICM patients (Table 3) [55, 56, 57, 58]. A multicenter study investigated the efficacy of LBBAP in reversing PICM in patients with infra-nodal block who had previously been implanted with standard RV pacing leads [58]. LBBAP was successfully upgraded in 19 out of 20 patients, resulting in improved LVEF and LVESV over a 12-month follow-up period, with stable lead performance.

**Table 3.** **Upgrade to left bundle branch area pacing in patients with pacemaker induced cardiomyopathy**.

Study	Year	Design	Total number of patients	Number of patients upgraded to LBBAP	Follow-up period (months)	Baseline EF (%)	Post LBBAP EF (%)	Pre LBBAP QRS (ms)	Post LBBAP QRS (ms)	Clinical outcomes
Qian *et al*. [55]	2021	Retrospective observational	13	13	10	40	48	174	117	Improvement in NYHA (*p* < 0.01)
		Single arm								Decreased Pro-BNP
Yang *et al*. [58]	2021	Retrospective observational	20	19	12	36	51	176	120	Improvement in NYHA (*p* = 0.02)
		Single arm								
Rademakers *et al*. [56]	2022	Retrospective observational	20	20	44	32	47	193	130	Improvement in NYHA (*p* < 0.01)
		Single arm								
Shan *et al*. [57]	2023	Retrospective observational	102	70	12	36	51	149	123	Improvement in NYHA (*p* < 0.001)

BNP, brain natriuretic peptide; EF, ejection fraction; LBBAP, left bundle branch area pacing; NYHA, New Work Heart Association.

## 7. Prevention

### 7.1 Strategies to Reduce Pacing Burden by Pacemaker Programming

Minimizing right ventricular pacing is advisable in patients without complete atrioventricular block. This can be accomplished through AAI pacing, setting the lowest clinically appropriate backup ventricular pacing rate, implementing a prolonged atrioventricular delay to facilitate intrinsic AV conduction, avoiding rate response pacing in individuals with a competent sinus node, and programming rate response settings that do not lead to non-physiological heart rates at rest or during activity. Nevertheless, demonstrating a significant improvement in clinical outcomes has proven to be challenging. In a meta-analysis involving 4119 patients, dual-chamber pacing programmed with algorithms to reduce RV pacing was compared to standard DDD pacing [59]. Despite the significant reduction in RV pacing burden through these algorithms, there were no discernible differences in terms of all-cause mortality, heart failure hospitalization, or the development of atrial fibrillation.

### 7.2 Exploring Other Pacing Sites

Initially, there was a hypothesis that decreasing the paced QRS duration via RV septal pacing would lower the occurrence of PICM. However, clinical trials that compared RV septal pacing to RV apical pacing did not reveal any clinical advantages for RV septal pacing. In the Protect-Pace study [60], there were no notable distinctions in terms of mortality, heart failure hospitalization, the prevalence of AF, or pro-brain natriuretic peptide (pro-BNP) levels between RV apical pacing and pacing in the high septal region.

### 7.3 Strategies Involving Biventricular Pacing Cardiac Resynchronization Therapy

Small comparative studies such as the Homburg Biventricular Pacing Evaluation (HOBIPACE) study [61] and COMBAT [62], which compared RV pacing to BiV-CRT in patients requiring pacing with reduced EF, demonstrated the superiority of BiV-CRT over RV pacing in enhancing cardiac function and quality of life in those with advanced LV dysfunction. In the Block-HF study, focused on patients with EF <50%, BiV-CRT outperformed RV pacing in reducing composite endpoints, primarily driven by heart failure hospitalization, without discernible differences in cardiac mortality [9]. However, the PACE trial, involving patients with normal EF, revealed that BiV-CRT prevented LV remodeling and a reduction in EF but found no differences in functional parameters or heart failure hospitalization [63]. The BioPACE trials yielded conflicting results regarding the benefit of BiV-CRT in patients with relatively preserved EF (>40%), demonstrating no mortality benefit and uncertain effects on hospitalization [8, 64]. All aforementioned studies comparing BiV-CRT to RV pacing in patients with preserved LV function failed to show any advantages in terms of mortality or hospitalization due to heart failure. According to current guidelines [65], BiV pacing is recommended in patients with an EF of 36–50% who develop AV block and are expected to require >40% RV pacing. However, caution is advised when EF is >50%, considering mixed results from the PACE and BioPACE trials. Routine BiV-CRT implantation in patients likely to experience a high burden of RV pacing has not become a standard of care unless these patients have pre-existing LV dysfunction.

### 7.4 Strategies Involving Conduction System Pacing

Conduction system pacing, which includes HBP or LBBAP, holds great promise as it may avoid the risk of PICM while allowing for the implantation of a dual chamber pacing system. In an observational investigation, the initial placement of HBP, in contrast to a standard dual-chamber pacemaker, was associated with a notable decrease in the composite endpoint comprising death, heart failure-related hospitalization, or the need for an upgrade to BiV-CRT [50]. Additionally, there was a trend toward reduced mortality with HBP. However, HBP does have its limitations, including elevated pacing thresholds, sensing issues, and difficulties in device programming. Recently, LBBAP has emerged as a promising alternative to address the shortcomings of HBP and is increasingly being utilized for both bradycardia and heart failure. The findings from a large European multicenter registry study (MELOS study) [66] demonstrated that LBBAP is a viable primary pacing method for bradyarrhythmia, achieving an overall success rate of 92.4% with a complication rate of 8.3% which includes peri-procedural chest pain (2.5%), acute perforation of the left ventricle (3.6%), lead dislodgement (1.5%), and pacing threshold issues (0.7%). Consequently, a recent European survey indicated that clinicians are increasingly favoring LBBAP over HBP as their first-line approach for bradyarrhythmia [67]. However, it’s worth noting that the bulk of evidence regarding the safety and efficacy of these techniques is derived from observational studies, and the long-term safety of LBBAP remains uncertain. Currently, several global clinical trials are underway to investigate the efficacy and safety of LBBAP, including PROTECT-HF (Physiological *vs.* Right Ventricular Pacing Outcome Trial Evaluated for bradyCardia Treatment, NCT05815745), PROTECT-SYNC (Preventive Effect of Left Bundle Branch Area Pacing Versus Right Ventricular Pacing on all Cause death, Heart Failure Progression, and Ventricular dyssynchrony in Patients with Substantial Ventricular Pacing, NCT05585411), OptimPacing (Protection of Cardiac Function with Left Bundle Branch Pacing in Patients with Atrioventricular Block, NCT04624763) and LEAP-Block (Impact of Left Bundle Branch Area Pacing *vs.* Right Ventricular Pacing in Atrioventricular Block, NCT04730921). Parameters such as the stability of capture thresholds, lead integrity, and lead extractability will need to be assessed in adequately powered studies with extended follow-up periods.

## 8. Conclusions

PICM is a common complication that can arise from permanent RV pacing. It is characterized by a reduction in EF in cases of chronic RVP, and constitutes just one aspect of PICM. After pacemaker implantation, many patients may experience the onset of new heart failure symptoms or atrial fibrillation. While some individuals can endure RV pacing for prolonged periods without apparent adverse effects, there is considerable variability in susceptibility to the detrimental effects of RV pacing. Further research is necessary to identify those at higher risk of developing PICM and tailor preventive measures accordingly. Current strategies for managing PICM encompass BiV-CRT and CSP, both associated with enhanced LV systolic function and improved clinical outcomes. However, the routine adoption of BiV-CRT and CSP implantation in patients expected to undergo a high burden of RV pacing has not become standard practice, except for those with pre-existing LV dysfunction. Randomized trials comparing the clinical outcomes of CSP with conventional implantation methods are needed to establish guidelines for its routine adoption.
